# Simultaneous Quantification of Mitochondrial Mature Frataxin and Extra-Mitochondrial Frataxin Isoform E in Friedreich’s Ataxia Blood

**DOI:** 10.3389/fnins.2022.874768

**Published:** 2022-04-28

**Authors:** Qingqing Wang, Laurent Laboureur, Liwei Weng, Nicolas M. Eskenazi, Lauren A. Hauser, Clementina Mesaros, David R. Lynch, Ian A. Blair

**Affiliations:** ^1^Center of Excellence in Environmental Toxicology, Department of Systems Pharmacology and Translational Therapeutics, Perelman School of Medicine, University of Pennsylvania, Philadelphia, PA, United States; ^2^Penn/CHOP Center of Excellence in Friedreich’s Ataxia, Philadelphia, PA, United States; ^3^Departments of Pediatrics and Neurology, Children’s Hospital of Philadelphia, Philadelphia, PA, United States; ^4^Departments of Pediatrics and Neurology, Perelman School of Medicine, University of Pennsylvania, Philadelphia, PA, United States

**Keywords:** assay validations, mass spectrometry, stable isotope dilution, neurodegenerative disease, liquid chromatography, homozygous Friedreich’s ataxia, heterozygous Friedreich’s ataxia

## Abstract

Friedreich’s ataxia (FRDA) is an autosomal recessive disease caused by an intronic guanine-adenine-adenine (GAA) triplet expansion in the frataxin (*FXN*) gene, which leads to reduced expression of full-length frataxin (1–210) also known as isoform 1. Full-length frataxin has a mitochondrial targeting sequence, which facilitates its translocation into mitochondria where it is processed through cleavage at G^41^-L^42^ and K^80^-S^81^ by mitochondrial processing (MPP) to release mitochondrial mature frataxin (81–210). Alternative splicing of *FXN* also leads to expression of N-terminally acetylated extra-mitochondrial frataxin (76–210) named isoform E because it was discovered in erythrocytes. Frataxin isoforms are undetectable in serum or plasma, and originally whole blood could not be used as a biomarker in brief therapeutic trials because it is present in erythrocytes, which have a half-life of 115-days and so frataxin levels would remain unaltered. Therefore, an assay was developed for analyzing frataxin in platelets, which have a half-life of only 10-days. However, our discovery that isoform E is only present in erythrocytes, whereas, mature frataxin is present primarily in short-lived peripheral blood mononuclear cells (PBMCs), granulocytes, and platelets, meant that both proteins could be quantified in whole blood samples. We now report a quantitative assay for frataxin proteoforms in whole blood from healthy controls and FRDA patients. The assay is based on stable isotope dilution coupled with immunoprecipitation (IP) and two-dimensional-nano-ultrahigh performance liquid chromatography/parallel reaction monitoring/high resolution mass spectrometry (2D-nano-UHPLC-PRM/HRMS). The lower limit of quantification was 0.5 ng/mL for each proteoform and the assays had 100% sensitivity and specificity for discriminating between healthy controls (*n* = 11) and FRDA cases (*N* = 100 in year-1, *N* = 22 in year-2,3). The mean levels of mature frataxin in whole blood from healthy controls and homozygous FRDA patients were significantly different (*p* < 0.0001) at 7.5 ± 1.5 ng/mL and 2.1 ± 1.2 ng/mL, respectively. The mean levels of isoform E in whole blood from healthy controls and homozygous FRDA patients were significantly different (*p* < 0.0001) at 26.8 ± 4.1 ng/mL and 4.7 ± 3.3 ng/mL, respectively. The mean levels of total frataxin in whole blood from healthy controls and homozygous FRDA patients were significantly different (*p* < 0.0001) at 34.2 ± 4.3 ng/mL and 6.8 ± 4.0 ng/mL, respectively. The assay will make it possible to rigorously monitor the natural history of the disease and explore the potential role of isoform E in etiology of the disease. It will also facilitate the assessment of therapeutic interventions (including gene therapy approaches) that attempt to increase frataxin protein expression as a treatment for this devastating disease.

## Introduction

Friedreich’s ataxia (FRDA) is an autosomal recessive neurodegenerative disorder caused by the deficient expression of mitochondrial mature frataxin protein. FRDA is (Marmolino, 2011) considered a rare disease, as it has an estimated prevalence of one in 50,000 in the Caucasian population (Delatycki et al., 2000), which makes it the most common hereditary ataxia in this population. FRDA is characterized by progressive sensory ataxia, areflexia, dysarthria, loss of position and vibratory sense and extensor plantar response (Lynch and Seyer, 2014). As a result of its progressive nature, most patients are wheelchair bound by 15.5 ± 7.4 years (mean age ± SD) after the onset of disease (Harding, 1981; Evans-Galea et al., 2014). However, heart disease is the major cause of death, which typically occurs in the fourth decade of life (Pousset et al., 2015). At present, there is no effective treatment for FRDA, although omaveloxolone was found to improve neurological function, when compared to placebo, and so it provides a potential future therapeutic strategy (Lynch et al., 2021).

The genetic basis of most cases (approximately 97%) is a homozygous guanine-adenine-adenine (GAA) triplet repeat expansion in the first intron of the frataxin (*FXN)* on both alleles (GAA1 and GAA2) (Chamberlain et al., 1988; Campuzano et al., 1996), which causes epigenetic transcriptional silencing and reduced expression of full-length frataxin protein (Chamberlain et al., 1988; Santos et al., 2010). A minority of FRDA patients (<3%) are compound heterozygotes possessing point or small mutations in the non-expanded allele and a GAA repeat expansion in the other (Cossee et al., 1999; Gellera et al., 2007). This leads to a loss of functional protein by non-transcriptional mechanisms. In typical FRDA patients, the length of the shortest GAA expansion (GAA1) correlates with the severity of the disease and conversely correlates with the age of onset; longer GAA expansions result in earlier onset and a faster progression (Filla et al., 1996; Sacca et al., 2011b), The presence of compound heterozygotes can complicate confirmation of a clinical diagnosis (Anheim et al., 2012) and the phenotype of compound heterozygotes cannot be predicted with certainty (Forrest et al., 1998).

**FIGURE 1:**
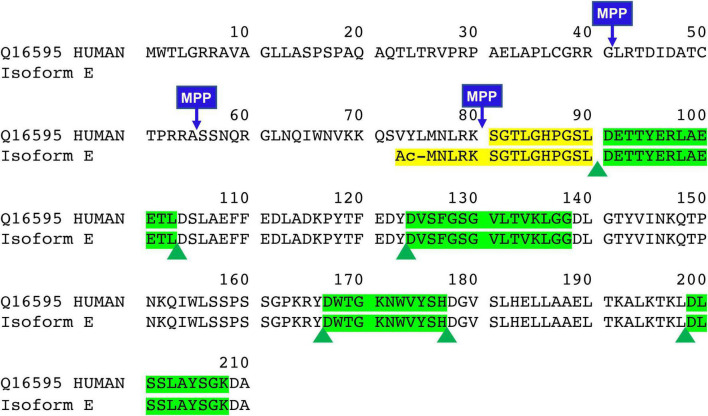
Sequence of full-length, intermediate, mature form, and new isoform of frataxin (FXN isoform E). Green highlights are quantitative peptides after Asp-N digestion which represent the total amount of frataxin in the whole blood. Yellow highlights are unique Asp-N digested peptide in mature form (SGTLGHPGSL) and frataxin isoform E (Ac-MNLRKSGTLGHPGSL).

Human full-length frataxin (isoform 1, MW = 23,135 Da) is expressed as a 210 amino acid precursor protein with a N-terminal mitochondrial targeting sequence (Figure 1). A two-step proteolytic cleavage by mitochondrial processing peptidase (MPP) result in the initial formation of an intermediate form (42–210; MW 18,826 Da), which is then converted to mitochondrial mature frataxin (81–210; MW = 14,268 Da) (Gakh et al., 2002; Condo et al., 2007; Schmucker et al., 2008). In contrast, extra-mitochondrial frataxin E protein (Figure 1; 76–210; MW = 14,953) in erythrocytes lacks a mitochondrial targeting sequence and arises through alternative splicing followed by N-terminal acetylation during translation (Guo et al., 2018a). No function has yet been ascribed to frataxin isoform E, and the exact function of mature frataxin has not yet been completely defined. However, several lines of evidence strongly suggest that mature frataxin is a functional component in a series of pathways including iron-sulfur cluster assembly, iron storage, heme biosynthesis, the respiratory chain, and cellular response to oxidative stress (Campuzano et al., 1996; Marmolino, 2011).

Frataxin levels have been measured by western blot analysis, electro-chemiluminescence (ECL), enzyme-linked immunosorbent assay (ELISA), and western blot from FRDA lymphoblasts, lymphocytes, and peripheral blood mononuclear cells (PBMCs) (Table 1) (Campuzano et al., 1997; Boesch et al., 2007; Boehm et al., 2011). However, these studies were limited by small samples sizes and/or by their time-consuming nature. Therefore, lateral flow immunoassay (LFI), was developed as a sensitive, rapid and accurate method for quantification of frataxin from a variety of cell types (Table 1; Deutsch et al., 2010; Sacca et al., 2011b; Plasterer et al., 2013). There is no need to purify mitochondria allowing fast one-step sample preparation and rapid assay performance (normally less than 45 min). Another immunoassay-based method for frataxin with multiplex capabilities using Luminex xMAP^®^ technology was developed and validated (Deutsch et al., 2014). The nature of the Luminex assay allows multiple purposes for the investigation of multiple markers of disease or markers for multiple diseases from a single sample. Only a small amount of blood is required for the Luminex assay and dried blood spots were typically used for frataxin determination. In most studies, the relative frataxin levels reported show consistently reduced levels in all types of specimens compared to controls (mean = 28.6% of controls, Table 1). Interestingly, a large variation of frataxin levels was observed in heterozygous FRDA patients compared to homozygous FRDA patients (Sacca et al., 2011b; Deutsch et al., 2014; Lazaropoulos et al., 2015; Clark et al., 2017). This was most likely due to cross-reaction of the antibody with the increased levels of isoform E found in some of the heterozytes (Guo et al., 2018a).

**TABLE 1:** Clinical studies of frataxin levels.

Method		Control		Carriers			Cases			References
	Sample	*n*	Level	*n*	Level	CTL (%)	*n*	Level	CTL (%)	
LFI	L-blast	5	438 pg/μg	5	281 pg/μg	64.0	5	127 pg/μg	29.0	Willis et al., 2008
ECL	L-blast	5	9.1 pg/μg	NC	NC	NC	11	3.0 pg/μg	33.0	Steinkellner et al., 2010
LFI	Buccal	40	30.0 pg/μg	81	15.5 pg/μg	50.5	195	6.3 pg/μg	21.1	Sacca et al., 2011b
LFI	PBMC	29	38.6 pg/μg	33	26.5 pg/μg	68.7	241	13.8 pg/μg	35.0	Deutsch et al., 2010
LFI	PBMC	19	41.3 pg/μg	31	25.3 pg/μg	61.3	12	12.7 pg/μg	30.8	Sacca et al., 2011a
ELISA	Lymph	50	0.11 pg/μg	NC	NC	NC	NC	NC	NC	Boehm et al., 2011
LFI	Lymph	9	23.0 AU/μg	NC	NC	NC	10	9.0 AU/μg	39.1	Selak et al., 2011
LFI	Platelet	9	9.4 AU/μg	NC	NC	NC	9	3.2 AU/μg	34.0	Selak et al., 2011
LFI	Blood	4	55.0 ng/mL	NC	NC	NC	31	15.0 ng/mL	27.3	Plasterer et al., 2013
LFI	PBMC	4	7.0 pg/μg	NC	NC	NC	31	3.0 pg/μg	42.0	Plasterer et al., 2013
LFI	Buccal	NC	NC	NC	NC	NC	31	1.5 pg/μg	NC	Plasterer et al., 2013
xMAP	Blood	319	33.0 ng/mL	63	15.0 ng/mL	45.5	117	5.0 ng/mL	15.2	Oglesbee et al., 2013
LFI	Blood	67	NP	143	NP	67.8	246	NP	26.1	Deutsch et al., 2014
LFI	Buccal	93	NP	271	NP	56.9	288	NP	19.7	Deutsch et al., 2014
xMAP	Blood	323	28.5 ng/mL	76	15.2 ng/mL	53.3	117	5.6 ng/mL	19.6	Deutsch et al., 2014
WB	Buccal	306	NP	NC	NC	NC	119	NP	NP	Lazaropoulos et al., 2015
WB	Blood	306	NP	NC	NC	NC	119	NP	NP	Lazaropoulos et al., 2015
ECL	PBMC	NC	NC	NC	NC	NC	24	0.4 pg/μg	NP	Yiu et al., 2015
LC-MS	Platelet	7	9.4 pg/μg	NC	NC	NC	7	2.4 pg/μg	28.7	Guo et al., 2018b
LC-MS	Blood	11	7.1 ng/mL	NC	NC	NC	45	2.1 ng/mL	29.6	Guo et al., 2018a
	**Total**	**828**		**289**	**Mean**	**58.5**	**815**	**Mean**	**28.6**	

*WB, Whole blood, AU, absorbance unites, NC, Not collected, NA, Not available NP, not provided; lymph, lymphocyte, L-blast, lymphoblast, μg, μg protein.*

In contrast to relative levels, absolute frataxin levels showed considerable differences between laboratories. A mean frataxin levels of 438 pg/μg (*n* = 5) was reported in control lymphoblasts from one study (Willis et al., 2008) while another study reported a mean level using an ECL assay in the same cells that was almost 30-times lower at 11.9 pg/μg (*n* = 5) (Steinkellner et al., 2010; Table 1). The concentration of frataxin in normal erythrocytes was reported to be 78.6 ng/mL or 85% of the whole blood frataxin level using LFI (Plasterer et al., 2013). In contrast, a level of 28.5 ng/mL was reported from dried blood spots by Luminex xMAP (Deutsch et al., 2014). Unfortunately, few of the studies have reported details of recombinant frataxin used to construct the standard curves (Steinkellner et al., 2010; Boehm et al., 2011; Oglesbee et al., 2013) and even fewer studies have performed vigorous method validation before clinical sample analysis (Oglesbee et al., 2013; Deutsch et al., 2014). Importantly, different forms of frataxin could not be distinguished by any of these assays so they would quantify both mitochondrial and extra-mitochondrial forms (Nachbauer et al., 2011).

**FIGURE 2:**
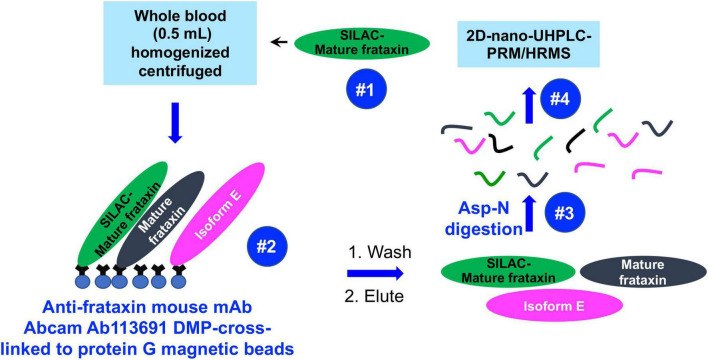
Schematic showing IP of frataxin isoforms from whole blood and analysis by 2D-nano-UHPLC-PRM/MS.

Mass spectrometry (MS) has the potential to discriminate different isoforms of frataxin protein by carefully choosing signature peptides which are released from different isoforms. We previously reported a sensitive assay of quantifying mitochondrial mature frataxin (81–210) from human platelets using immunoprecipitation (IP) coupled with two dimensional-nano-ultrahigh performance liquid chromatography-parallel reaction monitoring (2D-nano-UHPLC-PRM)/MS (Guo et al., 2018b). The characterization of isoform E as the frataxin proteoform that is present only in long-lived erythrocytes (Guo et al., 2018a) has now made it possible to use whole blood (rather than much less convenient blood platelets) for frataxin quantification. We report a validated, sensitive, specific, and robust assay for simultaneous determination of the two isoforms in blood using IP coupled with Asp-N endoproteinase to cleave peptide bonds on the N-terminal side of aspartic acid residues, followed by 2D-nano-UPLC-PRM/MS analysis of selected Asp-N peptides (Figure 2). The method employed stable isotope labeling by amino acids in cell culture (SILAC)-labeled human mature frataxin as the internal standard.

## Experimental Section

### Chemicals and Materials

All reagents and solvents were LC-MS grade quality unless otherwise noted. [^13^C_6_^15^N_2_]-lysine and [^13^C_6_^15^N_1_]-leucine were from Cambridge Isotope Laboratories (Andover, MA, United States). Anti-frataxin mouse monoclonal antibody [17A11] Ab113691 was from Abcam (Cambridge, MA, United States). Dimethyl pimelimidate dihydrochloride (DMP), Ethylenediaminetetraacetic acid (EDTA), cOmplete™ Mini EDTA-free Easypack protease inhibitor cocktail tablets, endoproteinase Asp-N sequencing grade, DL-dithiothreitol (DTT), bovine serum albumin (BSA), human lysozyme, imidazole, glycerol, phenylmethylsulfonyl fluoride (PMSF), triethanolamine, ethanolamine, and M9, minimal salts, 5X powder, minimal microbial growth medium (M9 media) were purchased from MilliporeSigma (Billerica, MA, United States). Ni-NTA agarose resin was purchased from Qiagen (Germantown, MD, United States). LC grade water and acetonitrile were from Burdick and Jackson (Muskegon, MI, United States). Ammonium bicarbonate and acetic acid were purchased from Fisher Scientific (Pittsburgh, PA, United States). Protein G magnetic beads were obtained from Life Technologies Corporation (Grand Island, NY, United States).

### Clinical Samples

Blood samples were obtained from 11-unaffected healthy control subjects and 100-homozygous FRDA patients (Table 2) and 6-heterozygous FRDA patients (Table 3). A blood pool was made from 6 FRDA patients with > 800 GAA1 repeat (Supplementary Table 1). All were enrolled in parallel in an ongoing natural history study at the Children’s Hospital of Philadelphia (Lynch et al., 2006). Written informed consent was obtained from each donor participating in the study. If subjects were under the age of 18, written informed consent was obtained from a parent and/or legal guardian. The study was approved by the Institutional Review Board (IRB) of the Children Hospital of Philadelphia (IRB Protocol # 01–002609). Venous blood was drawn in 8.5 mL purple cap Vacutainer EDTA tubes and gently invert to mix. All samples were immediately aliquoted to Eppendorf tubes and frozen at −80^°^C until analysis.

**TABLE 2:** Demographics for study cohort of healthy controls and study cohort of homozygous FRDA patients.

Subject	Total	Male	Female	GAA1 mean	Age of onset mean
Healthy controls	11	5	6	NA	NA
FRDA patients	100	40	60	744	8.8

**TABLE 3:** Demographics for study cohort of heterozygous FRDA patients.

Mutation	Symbol	GAA-1	GAA-2	Onset age	M/F
L106S	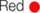	832	c.317 T > C	3	M
Aberrant splicing	Brown 🌑	900	g.1 1-5 G > C	3	M
M1S	green ■	Unknown	c.2 del T	7	M
A34P	blue ■	733	c.100 del G	22	M
G130V	cyan ▲	1150	p.389 G > T	13	M
Deletion	black 🌑	566	Deletion	7	F

### Expression and Purification of Unlabeled and Stable Isotope Labeling by Amino Acids in Cell Culture-Labeled Mature Frataxin

The expression of unlabeled and SILAC-labeled mature frataxin was performed in *Escherichia coli* BL21 DE3 as described previously (Guo et al., 2018b). Briefly, the coding sequence of human mature frataxin (81–210) was amplified from *FXN* cDNA plasmid (pTL1), then cloned into a pET21b plasmid and linked to the 6× histidine (His) sequence. The 6× His-tag fusion of frataxin was expressed in *Escherichia coli* BL21 DE3 in M9 media containing 1 mM MgSO_4_, 10 μM CaCl_2_, and 0.5% glucose with 100 mg/L ampicillin. For expressing unlabeled frataxin, the M9 medium was supplemented with 0.025% leucine and lysine. For expressing SILAC-labeled frataxin, the M9 medium was supplemented with 0.025% [^13^C_6_^15^N_1_]-leucine and [^13^C_6_^15^N_2_]-lysine. The cell pellets were collected and lysed in lysis buffer [50 mM Tris-HCl (pH 8.0), 500 mM NaCl, 10 mM imidazole, 10% glycerol, 2 mM β-mercaptoethanol, 2× protease inhibitor mix, 1 mM PMSF] containing 100 μg/mL human lysozyme. The lysate was centrifuged at 20,000 × *g* for 30 min at 4^°^C, and the supernatant was purified with Ni-NTA resin. The purity of the unlabeled mature frataxin and SILAC-labeled mature frataxin was confirmed to be > 95% by SDS-PAGE and Coomassie blue staining.

### Whole Blood Sample Preparation Before Immunoprecipitation

All blood samples were thawed at room temperature, and 500 μL of each sample was mixed with 750 μL NP-40 lysis buffer (150 mM NaCl, 50 mM Tris/HCl pH 7.5, 0.5% Triton X-100, 0.5% NP-40, 1 mM DTT, 1 mM EDTA) containing protease inhibitor cocktail. The same amount of SILAC-labeled mature frataxin (20 ng) was spiked in each sample (calibrator, QC and whole blood) as an internal standard. Samples were lysed by probe sonication on ice for 30 pulses at power 5 using a sonic dismembranator (Fisher, Pittsburgh, PA, United States), followed by centrifugation at 17,000 *g* for 15 min at 4^°^C. The supernatant was transferred from the pellet and incubated with pre-made DMP-crosslinked anti-frataxin protein G beads for immunoprecipitation.

### Immunoprecipitation and Asp-N Digestion

Mouse monoclonal anti-frataxin antibody (4 μg) was cross-linked to protein G beads (0.5 mg) through DMP as described previously (Guo et al., 2018b). Briefly, mouse monoclonal anti-frataxin antibody was firstly incubated with protein G beads overnight at 4^°^C to form the antibody coupled protein G beads. The antibody coupled beads were incubated with 13 mg/mL DMP solution 1 h at room temperature to form the stable cross-linked anti-frataxin protein G beads. The cross-linked protein G beads can be kept in PBS at 4^°^C for a week.

The processed whole blood samples (1.25 mL) were added into 0.5 mg anti-frataxin protein G beads to carry out immunoprecipitation at 4^°^C overnight under rotary agitation. The beads were washed with PBS with 0.02% Tween-20 three times and frataxin and its isoforms were eluted with 100 μL of elution buffer (90% 100 mM acetic acid/10% acetonitrile). Elutes were transferred to deactivated glass inserts (Waters, Milford, MA, United States) and dried in a vacuum concentrator (Jouan RC 10.22, Fisher, Pittsburgh, PA, United States). Samples were dissolved in 50 μL of 25 mM aqueous ammonium bicarbonate containing 100 ng of Asp-N and incubated at 37 overnight before LC-MS analysis.

### Method Validation by Stable Isotope Labeling by Amino Acids in Cell Culture-Labeled Mature Frataxin

A 5% BSA and FRDA whole blood pool from six FRDA patients (Supplementary Table 1) were used for preparation of calibration standards and quality controls (QCs). Calibration standards and QCs were prepared in two types of matrix, 500 μL of 5% BSA and 500 μL of FRDA whole blood pool for the evaluation of matrix effect. In 5% BSA, calibration standards (0.5, 2.0, 4.0, 10.0, 20.0, 40.0, and 80.0 ng/mL) and QCs (1.5, 3.0, 30, and 60 ng/mL) were prepared from spiking in additional stock solutions of mature frataxin at 2, 0.2, or 0.02 ng/mL. In FRDA blood pool, calibration standards (baseline + 0, 2.0, 4.0, 10.0, 20.0, 40.0, and 80.0 ng/mL) and QCs (baseline + 0, 3.0, 30, and 60 ng/mL) were prepared from spiking in additional stock solutions of mature frataxin at 2, 0.2, or 0.02 ng/mL. The extra spiked volume was less than 90% of the matrix. The accuracy and precision were determined on four levels of QCs, lower limit of quantification (LLOQ, 1.5 ng/mL), low QC (LQC, 3.0 ng/mL), middle QC (MQC, 30 ng/mL), and high QC (HQC, 80 ng/mL) in 5% BSA. The QCs were prepared in pooled FRDA blood where the frataxin level had been determined. This value was subtracted from the values that were obtained. The intra-batch accuracy and precision were determined on QCs (*n* = 5) were performed and analyzed on the same day. The inter-batch accuracy and precision were determined on QCs (*n* = 3) were performed and analyzed on three different days. The acceptance criteria of ± 15% for calibration stands and QCs (± 20% at lowest QC) at protein level were applied.

### Stability Assessment

Frataxin stability in whole blood at room temperature was evaluated in healthy control whole blood over a 24-h period. After blood sample collection, 500 μL of blood were aliquoted to eight Eppendorf tubes. The aliquots were kept at room temperature on the benchtop for 1, 2, 4, 6, 8, 12, and 24 h, then frozen at −80^°^C until analysis. Room temperature stability was evaluated by comparing the incubated samples to freshly prepared sample (*T* = 0). The freeze-thaw stability was evaluated (−80°C/room temperature) with three replicates at MQC (*n* = 3, 30 ng/mL).

### 2D-Nano-UHPLC-PRM/MS

Mass spectrometry was conducted using a Q Exactive HF Orbitrap high resolution mass spectrometer coupled to Dionex Ultimate 3000 RSLCnano with capillary flowmeter chromatographic systems (Thermo Fisher Scientific, San Jose, CA, United States). The 2D system was setup as a pre-concentration mode which was composed of a 10-port valve, one nanopump and a micropump. The LC trapping column was an Acclaim PepMap C_18_ cartridge (0.3 μm × 5 mm, 100 Å, Thermo Scientific) and the analytical column was a C_18_ AQ capillary column with a 10 μm pulled tip (75 μm × 25 cm, 3 μm particle size; Columntip, New Haven, CT, United States). The 2D-nano-UHPLC system was controlled by Xcalibur software from the Q-Exactive mass spectrometer.

Loading solvent was water/acetonitrile (99.7:0.3; v/v) containing 0.2% formic acid. Solvent A was water/acetonitrile (99.5:0.5; v/v) containing 0.1% formic acid, and solvent B was acetonitrile/water (98:2, v/v) containing 0.1% formic acid. The valve stayed at loading position (1–2) with loading solvent on the trapping column at 10 μL/min for 4 min. Then the valve changed to injection position (1–10) at which the trapping column was connected with the analytical column, and samples were back-flushed into the analytical column. The valve was changed back to the loading position (1–2) at 50 min at which the trapping column was equilibrated with loading solvent and analytical column was equilibrated with 2% B. Samples were eluted with a linear gradient at a flow rate of 0.4 μL/min. The gradient on the analytical column was as follows: 2% B at the start, 5% B at 10 min, 20% B at 15 min, 60% B at 35 min, 80% B at 40–48 min, and 2% B at 48–60 min. Nanospray was conducted using Nanospray Flex*™* ion source (Thermo Scientific). MS operating conditions were as follows: spray voltage 2,500 V, ion transfer capillary temperature 275°C, ion polarity positive, S-lens RF level 55, in-source CID 2.0 eV. Both full scan and PRM were used. The full scan parameters were resolution 60,000, AGC target 2 e5, maximum IT 80 ms, scan range 350–1200 m/z. The PRM parameters were resolution 60,000, AGC target 2 e5, maximum IT 80 ms, loop count 5, MSC count 4, isolation window 1.0 m/z, NCE 25. The PRMs were scheduled for 20.5 to 22.5 min for S^81^GTLGHPGSL^90^, 24.0 to 28.0 min for D^124^VSFGSGVLTVKLGG^138^, 22.00–24.2 min for D^167^WTGKNWVYSH^177^, and 21.5–23.5 min for D^199^LSSLAYSGK^208^.

### Data Analysis

Data analysis was performed using Skyline (MacCoss Laboratory, University of Washington, Seattle, WA, United States). The peak area ratio of each PRM transition for each unlabeled/light (L) peptide to labeled/heavy (H) peptide was calculated by the Skyline software and used for absolute quantification. The peptide ratios were calculated by the average L/H ratios of the three PRM transitions. The total frataxin levels were calculated from the average of the four selected quantitative peptides, the mature frataxin levels were calculated from SGTLGHPGSL peptide, and the frataxin isoform E levels were calculated by subtracting SGTLGHPGSL peptide from total frataxin. Statistical analyses were conducted using Prism 9 for macOS Version 9.3.1 (GraphPad Software, LLC.). Comparisons between groups were made using an unpaired two-tailed *t*-test. Correlations between GAA repeats and frataxin levels as well as between age of onset and frataxin levels were conducted using linear regression models.

## Results

### Selection of Quantitative Peptides

**FIGURE 3:**
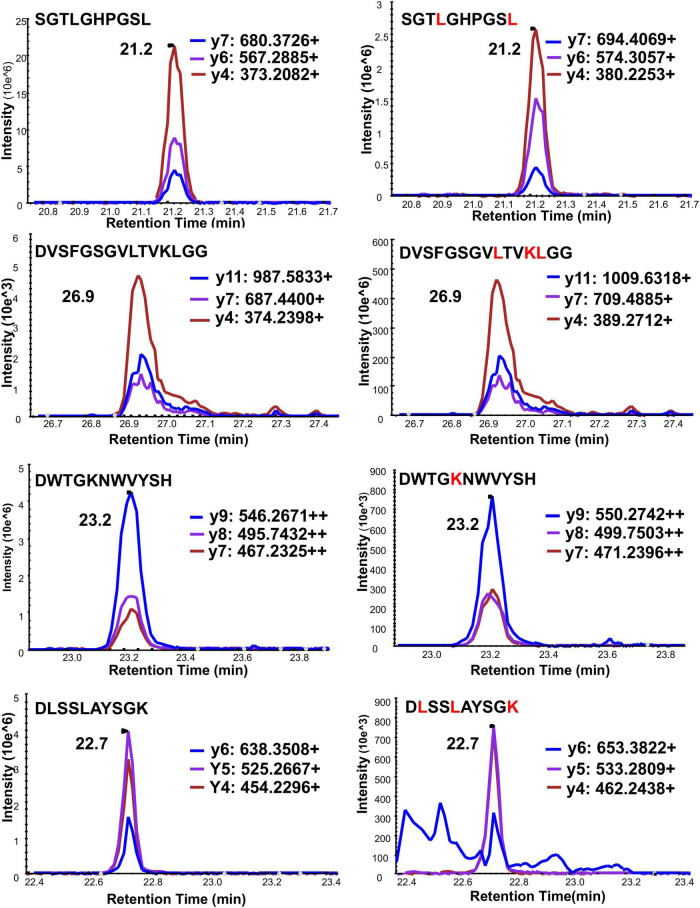
Typical chromatograms of three PRM ion transitions of four quantitative Asp-N peptides under PRM mode. Left panel is light peptide, right panel is heavy peptide. *Y*-axis is response intensity under PRM mode. *X*-axis is retention time (min). Three PRM transitions shown in blue, purple and red.

There is an arginine (R^79^) and a lysine (K^80^) before serine (S^81^) in the full-length frataxin sequence. Therefore, trypsin would potentially digest other all frataxin isoforms to release the same N-terminal tryptic S^81^GTLGHPGSLDETTYER^97^ peptide. In order to obtain the unique N-terminal peptides for differentiating mature frataxin and frataxin isoform E, Asp-N was chosen as the endoproteinase. Mature frataxin and frataxin isoform E release two different N-terminal peptides with Asp-N digestion, S^81^GTLGHPGSL^90^ and Ac-M^76^NLRKSGTLGHPGSL^90^ (Figure 1). The expressed and purified mature frataxin (81–210) and SILAC-labeled mature frataxin were digested by Asp-N and the proteolytic peptides were optimized under full scan and PRM/MS modes. The uniqueness of the Asp-N peptides was verified by using the human proteome downloaded from NIST as the background proteome. Initially, seven potentially useful proteolytic peptides with length at 5–12 amino acids were selected, and their precursor and all product ions were assessed. The three most intense Asp-N digested peptides chosen for quantifying total frataxin levels were: D^124^VSFGSGVLTVKLGG^138^, D^157^WTGKNWVYSH^177^, and D^199^LSSLAYSGK^208^ (Supplementary Table 2). The precursor ions exhibited 10–15-fold higher intensities than their corresponding product ions in the neat solution. However, the precursor peaks had interference peaks around or even not detected due to the high interfering background in the whole blood matrix. In contrast, product ions showed clean peaks as fewer interfering signals were present in the channels of product ions (Figure 3). Therefore, the three most intense product ions for corresponding peptides were used for quantifying both endogenous frataxin and SILAC-labeled mature frataxin in whole blood samples. Direct quantification of frataxin isoform E is not accurate since the oxidation of methionine happens during sample preparation step. Due to a variety of technical challenges (Arnesen, 2011; Aksnes et al., 2016), fully acetylated frataxin isoform E standard is not available. Therefore, isoform E levels were calculated by subtracting the amount of mature frataxin (determined from S^81^GTLGHPGSL^90^) from total frataxin determined as the mean of amounts determined from the three common peptides (D^124^VSFGSGVLTVKLGG^138^, D^157^WTGKNWVYSH^177^, and D^199^LSSLAYSGK^208^).

### Calibration Curves and Lower Limit of Quantitation

A 5% BSA and a whole blood pool from six FRDA patients (Supplementary Table 1) were used as a surrogate matrix and the biological matrix respectively in method validation. Calibration curves were constructed at six different concentrations ranging from 0.5 to 80.0 ng/mL in 5% BSA solution, and from baseline to baseline with spiked-in 80 ng/mL frataxin standard in the FRDA blood pool. Linear standard curves were obtained for each of the four peptides with r^2^ values range between 0.9986 to 0.9998 in 5% BSA matrix and between 0.9865 to 0.9983 in FRDA blood pool (Supplementary Figure 1). The LLOQ was set at 1.5 ng/mL in 5% BSA, which is below the mean concentration of total frataxin reported in whole blood from healthy controls and FRDA patients determined previously (Table 1).

### Accuracy and Precision

Method accuracy and precision were obtained for the analysis of four levels of QCs in whole blood for the LLOQ (1.5 ng/mL), LQC (3.0 ng/mL), MQC (30 ng/mL), and HQC (60 ng/mL). The intra-day precision (*N* = 5) for LLOQ, LQC, MQC, and HQC (Supplementary Table 3) and inter-day precision for LQC, MQC, and HQC (Supplementary Table 4) precision was better than ± 15% for total frataxin. The accuracy was better than 90–110%. Endogenous frataxin isoforms in the FRDA whole blood that was used to prepare QCs ere determined and subtracted from the determined amounts.

### Matrix Effect

A matrix effect could alter the absolute responses of the analyte and internal standard but would not change the L/H ratio of the response. Calibration curves generated from 5% BSA and from FRDA whole blood were compared (Supplementary Figure 1 and Supplementary Table 5). Two sets of calibration curves were parallel to each other. However, the matrix effect did not affect assay accuracy and precision of the assay.

### *In vitro* Sample Stability in Whole Blood

The frataxin levels in the whole blood were stable over a 24-h period at room temperature before sample preparation (Supplementary Table 6). The frataxin levels were the same after two freeze-thaw cycles in three replicates of whole blood (Supplementary Table 7). LQC, MQC, and HQC samples that were re-analyzed after 48 h standing in the autosampler (4^°^C) gave essentially identical data to that obtained from the original analyses (data not shown).

### Incurred Sample Reanalysis (ISR)

Twelve FRDA whole blood samples were re-analyzed. The deviations of the first analysis and repeat analysis for each individual for mature frataxin, isoform E and total frataxin ranged from −26.6 to 18.7% (Supplementary Table 8). The mean value of the first analysis and repeat analysis for mature frataxin, frataxin isoform E and total frataxin was −2.2, −13.3, and −10.8%, respectively (Supplementary Table 8). This demonstrated that data were reproducible in the whole blood samples.

### Frataxin Levels in Controls and Friedreich’s Ataxia Cases

**FIGURE 4:**
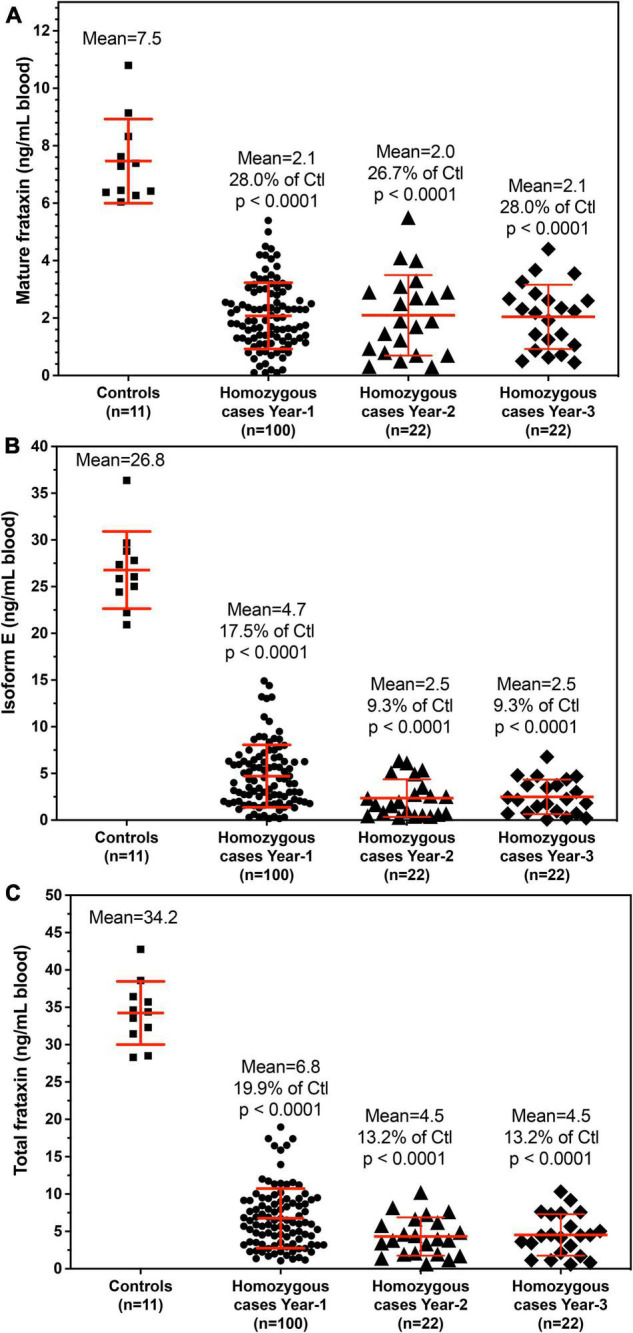
Whole blood frataxin levels in healthy controls and homozygous FRDA patients as means ± standard deviation. **(A)** Mature frataxin. Healthy controls 7.5 ± 1.5 ng/mL blood (*n* = 11), homozygous cases in year-1 2.1 ± 1.2 ng/mL blood (*n* = 100, *p* < 0.0001), homozygous FRDA patients in year-2 2.1 ± 1.4 ng/mL blood (*n* = 22, *p* < 0.0001), homozygous FRDA patients in year-3 2.0 ± 1.1 ng/mL blood (*n* = 22, *p* < 0.0001). **(B)** Frataxin isoform E. Healthy controls 26.8 ± 4.1 ng/mL blood (*n* = 11), homozygous FRDA patients in year-1 4.7 ± 3.3 ng/mL blood (*n* = 100, *p* < 0.0001), homozygous FRDA patients in year-2 2.5 ± 2.0 ng/mL blood (*n* = 22, *p* < 0.0001), homozygous FRDA patients in year-3 2.5 ± 1.9 ng/mL blood (*n* = 22, *p* < 0.0001). **(C)** Total frataxin. Healthy controls 34.2 ± 4.2 ng/mL blood (*n* = 11), homozygous FRDA patients in year-1 6.8 ± 4.0 ng/mL blood (*n* = 100, *p* < 0.0001), homozygous FRDA patients in year-2 4.5 ± 2.6 ng/mL blood (*n* = 22, *p* < 0.0001), homozygous FRDA patients in year-3 4.5 ± 2.8 ng/mL blood (*n* = 22, *p* < 0.0001). Homozygous FRDA patients were compared with healthy controls using an unpaired two-tailed *t*-test.

The demographic information for the homozygous and heterogenous FRDA patients enrolled in this study are summarized in Tables 2, 3. All frataxin levels were within the linear range of standard curves. The mean concentrations (± SD) of mature frataxin in healthy controls and homozygous FRDA cases in blood samples taken at year-1 were significantly different (*p* < 0.0001) at 7.5 ± 1.5 ng/mL and 2.1 ± 1.2 ng/mL, respectively (Figure 4A). The mean concentrations (± SD) of isoform E in healthy controls and homozygous FRDA cases in blood samples taken at year-1 were significantly different (*p* < 0.0001) at 26.8 ± 4.1 ng/mL and 4.7 ± 3.3 ng/mL, respectively (Figure 4B). The mean concentrations (± SD) of total frataxin in healthy controls and homozygous FRDA cases in blood samples taken at year-1 were significantly different (*p* < 0.001) at 34.2 ± 4.2 ng/mL and 6.8 ± 4.0 ng/mL, respectively (Figure 4C).

## Discussion

It has been reported that recombinant yeast and human frataxin are able to self-associate in large molecular assemblies as a mechanism for detoxifying redox-active iron (Cavadini et al., 2002; O’Neill et al., 2005). Therefore, to effectively release frataxin proteoforms from whole blood cells, different types of lysis buffers were screened. We found that the non-ionic detergents, NP-40 and Triton X-100 were effective and also less harsh than ionic detergents such as sodium dodecyl sulfate (SDS). Although these two detergents could potentially have affected chromatographic separations and contaminated the mass spectrometer, they were efficiently removed by washing the beads used for IP with PBS. This made it possible to quantify endogenous frataxin levels in whole blood from 11 controls and 100 FRDA patients using the validated stable isotope dilution 2D-nano-UHPLC-PRM/HRMS method. There was no overlap of mature frataxin, isoform E, or total frataxin levels between controls and homozygous FRDA patients using this assay (Figure 4). Therefore, the assay had 100% specificity and 100% sensitivity for distinguishing healthy controls from homozygous FRDA cases. Assays on a sub-set of patients conducted on samples obtained at year-2 and year-3 from the original year sampling were found to give very similar results for mature frataxin, isoform E and total frataxin (Figure 4). This confirmed that the assay is very robust and that the frataxin levels do not change significantly over a 2-year period. The levels of isoform E frataxin in whole blood from healthy controls were similar to those reported previously for frataxin in erythrocyte fractions obtained using a dipstick LFI (Plasterer et al., 2013) most likely because the dipstick assay could not distinguish mature frataxin from isoform E.

**FIGURE 5:**
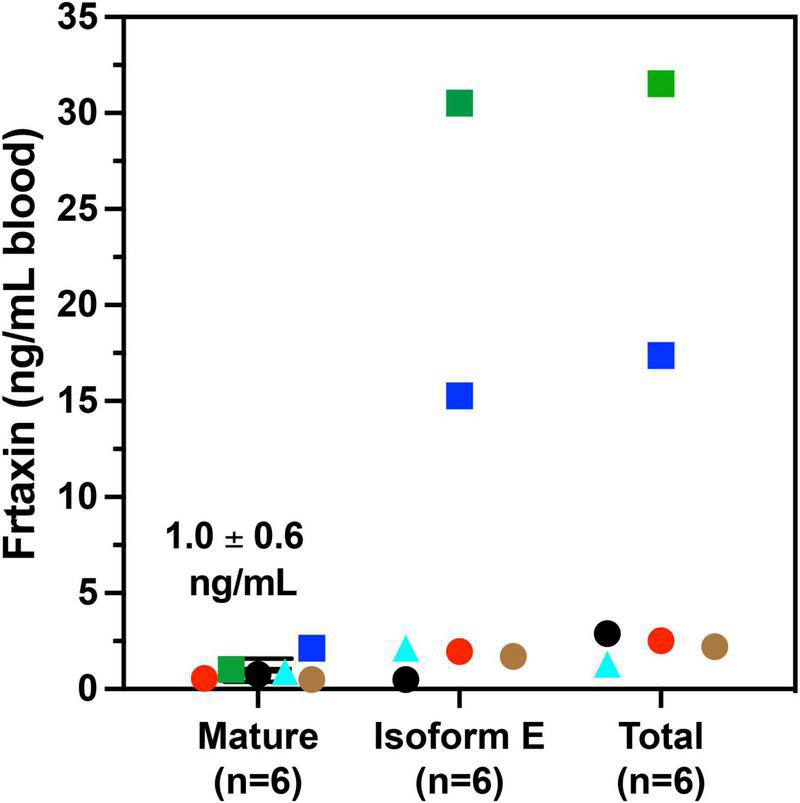
Whole blood frataxin levels in controls and compound heterozygous cases in year 1 (*n* = 6). 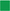 M1S (c.2 del T); 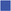 A34P (c.100 del G); 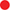 L106S (c.317 T > C); 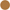 XX (g.1 1-5 G > C); 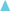 G130V (p.389 G > T); 🌑 unknown deletion.

A majority of FRDA patients (homozygous, 97%) carry GAA repeat expansions on both alleles, while a subgroup of patients carries a missense mutation on one allele and a GAA repeat expansion on the other (heterozygous, 3%). (Chamberlain et al., 1988; Campuzano et al., 1996). The phenotype of heterozygous patients cannot be predicted with certainty and the frataxin levels vary with specific point mutations. In our cohort, six of the patients were heterozygotes (Table 3). The c.317 T > C mutation (L106S) was reported as a type of missense mutation in the core of the protein, which was predicted to be a requirement for maintenance of a stable and compact frataxin structure (Dhe-Paganon et al., 2000). Missense mutations on this site could lead to folding abnormalities and yield essentially no protein (Lazaropoulos et al., 2015). In agreement with this prediction, a heterozygous FRDA subject with the L106S point mutation had lower levels of mature frataxin, isoform E, and total frataxin than expected based on the GAA1 repeat length of 832 (Figure 5, red circle). In contrast, the FRDA patient with a c.100delG point mutation (A34P) had higher levels of total frataxin, mature frataxin and frataxin isoform E than predicted based on a GAA1 repeat length of 733 (Figure 5, blue square). The c.100delG point mutation was found in exon 1 nt100, was predicted to cause a frameshift and premature termination of frataxin at codon 7 to theoretically give M1S at the amino terminus of the protein (Gellera et al., 2007). A heterozygous FRDA subject who carried a c.2delT mutation (M1S) had highly discrepant levels of frataxin between buccal cells (22% of controls) and whole blood (157% of controls) that were reported in a previous study (Lazaropoulos et al., 2015). The M1S mutation would result in a failure of initiation of translation and together with the effect of GAA1 repeat expansion reduced levels of mature frataxin would result (Zhu et al., 2002). We found a low level of mature frataxin (Figure 5 green square) for a subject with the same M1S point mutation. However, total frataxin was highly elevated because of the high levels of isoform E (Figure 5, green square). This subject was the only FRDA patient with total frataxin and frataxin isoform E levels, that fell into the range typically found for healthy controls (Figure 4).

The c.100 del G point mutation (A34P) resulted in levels of isoform E and total frataxin that were higher than was observed in most of the homozygous FRDA patients even though this patient had 733 GAA1 repeats (Figure 5, blue square). In contrast, the levels of mature frataxin were typical of those found in homozygous cases with this number of GAA1 repeats (Figure 5, blue square). Blood samples from the other three heterozygous FRDA patients that were analyzed including the patient with a c.100 del G mutation (G130V), had levels typical of homozygous FRDA cases. Furthermore, none of frataxin proteins that were analyzed in blood samples from the heterozygous FRDA cases had the mutations predicted from their mRNA sequences. Therefore, these proteins were either unstable and not present in the blood cells or they were lost during our IP procedure.

**FIGURE 6:**
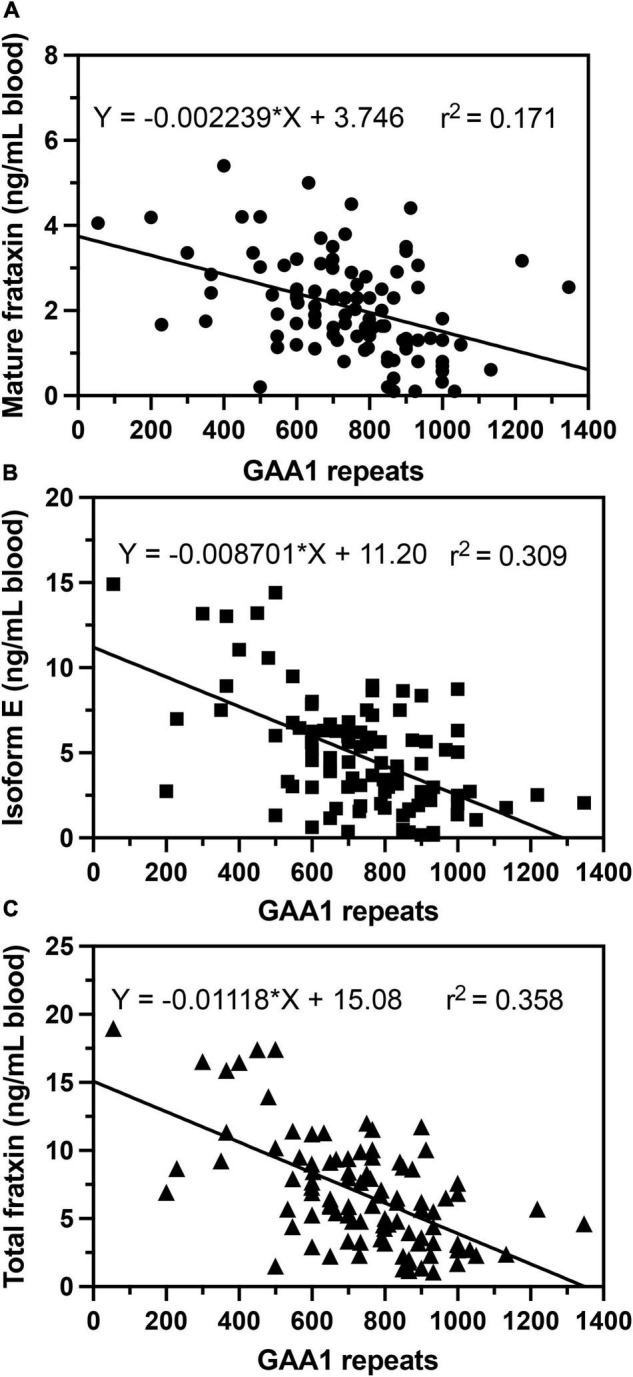
Correlations of GAA repeat length on the shorter allele (GAA1) with whole blood frataxin levels. **(A)** Mature frataxin (*p* < 0.0001). **(B)** Isoform E (*p* < 0.0001). **(C)** Total frataxin (*p* < 0.0001).

**FIGURE 7:**
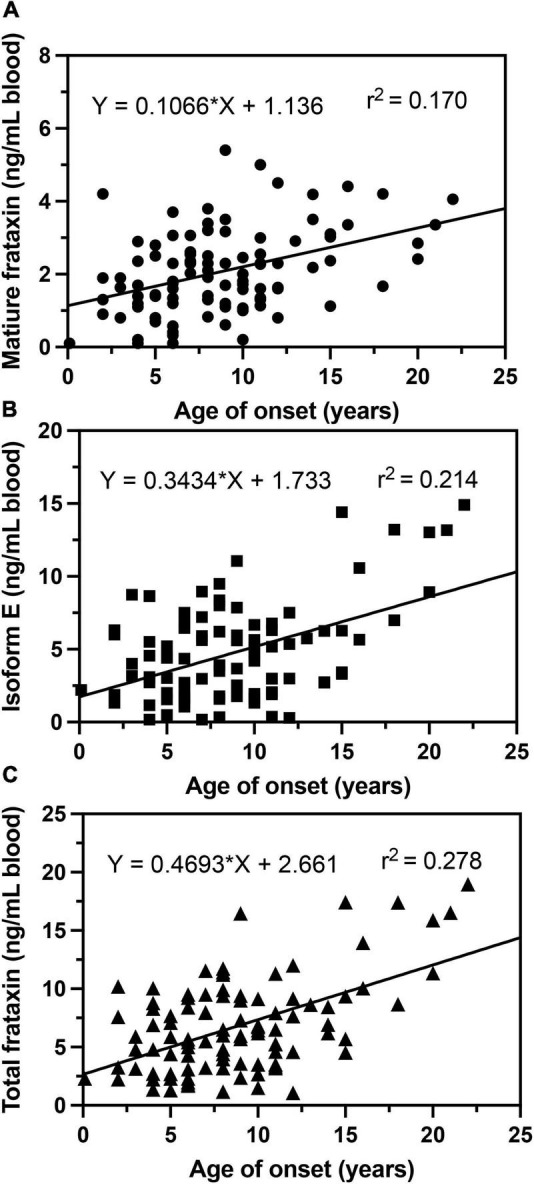
Correlations of age of onset with whole blood frataxin levels. **(A)** Mature frataxin (*p* < 0.0001). **(B)** Isoform E (*p* < 0.0001). **(C)** Total frataxin (*p* < 0.0001).

In agreement with previous studies (Filla et al., 1996; Deutsch et al., 2010; Plasterer et al., 2013), we observed that in homozygous FRDA patients, mature frataxin, isoform E, and total frataxin levels were inversely correlated with GAA1 repeat lengths (Figure 6). Furthermore, there a direct correlation between mature frataxin, isoform E, and total frataxin levels with age of onset of the disease (Figure 7). Therefore, the availability this validated robust, specific, and sensitive UHPLC-MS assay will make it possible to monitor mature frataxin, isoform E, and total frataxin levels for future studies on the natural history of the disease. It will also facilitate studies to explore the potential role of isoform E in etiology of the disease, particularly in heterozygous FRDA cases. Importantly, it will be possible with this assay to rigorously assess the efficacy of future therapeutic interventions (including gene therapy approaches) that attempt to increase frataxin protein expression as a treatment for this devastating disease.

## Data Availability Statement

The original contributions presented in the study are included in the article/Supplementary Material, further inquiries can be directed to the corresponding author.

## Ethics Statement

The studies involving human participants were reviewed and approved by the Children’s Hospital of Philadelphia. Written informed consent to participate in this study was provided by the participants’ legal guardian/next of kin.

## Author Contributions

QW, LL, LW, NE, and CM conducted the analyses. LH coordinated patient recruitment and sample acquisition. DL and IB directed the project and conceived the bioanalytical approach. QW, CM, DL, and IB drafted the original manuscript. All authors edited the final version of the manuscript.

## Conflict of Interest

The authors declare that the research was conducted in the absence of any commercial or financial relationships that could be construed as a potential conflict of interest.

## Publisher’s Note

All claims expressed in this article are solely those of the authors and do not necessarily represent those of their affiliated organizations, or those of the publisher, the editors and the reviewers. Any product that may be evaluated in this article, or claim that may be made by its manufacturer, is not guaranteed or endorsed by the publisher.
