# Supplementary material for: Simultaneous Quantification of Mitochondrial Mature Frataxin and Extra-Mitochondrial Frataxin Isoform E in Friedreich’s Ataxia Blood
Source: Front Neurosci. 2022 Apr 28;16:874768. doi: 10.3389/fnins.2022.874768 (PMC9098139; doi:10.3389/fnins.2022.874768)
Supplement: Supplementary file 1 [file Data_Sheet_1.pdf]

Simultaneous quantification of mitochondrial mature frataxin and extra-mitochondrial frataxin isoform E in Friedreich's ataxia blood: Supplementary Information

Table S1. FRDA patients' blood used for method validation

Patient ID	GAA1 repeats	GAA2 repeats	Age of onset	Sex
1	1000	1000	3	F
2	1000	1200	6	F
3	796	1129	15	F
4	603	1001	14	F
5	900	1200	6	M

Table S2. Quantitative Asp-N peptides and their three ion transitions for endogenous frataxin (light) and SILAC-labeled mature frataxin (heavy)

Peptide sequence	Precursor ion charge	Precursor Ion (m/z)	Product ion charge	Product ion	Product ion (m/z)
S81GTLGHPGSL90	2+	463.2405	1+	y7	680.3726
			1+	y6	567.2885
			1+	y4	373.2082
S81GT**L**GHPGS**L**90	2+	470.2577	1+	y7	694.4069
			1+	y6	574.2057
			1+	y4	380.2253
D124VSFGSGVLTVKLGG138	2+	718.3932	1+	y11	987.5833
			1+	y7	687.4400
			1+	y4	374.2398
D124VSFGSGV**L**TV**KL**GG138	2+	729.4175	1+	y11	1009.6318
			1+	y7	709.4885
			1+	y4	389.2712
D157WTGKNWVYSH177	3+	464.8826	2+	y9	546.2671
			2+	y8	495.7432
			2+	y7	467.2325
D157WTG**K**NWVYSH177	3+	467.5540	2+	y9	550.2742
			2+	y8	499.7503
			2+	y7	471.2396
D199LSSLAYSGK208	2+	520.7666	1+	y6	638.3508
			1+	y5	525.2667
			1+	y4	454.2296
D199**L**SS**L**AYSG**K**208	2+	531.7908	1+	y6	653.3822
			1+	y5	533.2809
			1+	y4	462.2438

K = [$^{13}C_6$,$^{15}N_2$]-lysine, **L**: = [$^{13}C_6$$^{15}N_1$]-leucine

Table S3. Intra-day precision and accuracy for determination of total frataxin using 5 replicates on a single day

LLOQ (1.5 ng/mL)

Peptide	Replicate1 (ng/mL)	Replicate2 (ng/mL)	Replicate3 (ng/mL)	Replicate4 (ng/mL)	Replicate5 (ng/mL)	Mean (ng/mL)	SD (ng/mL)	Precision (%)	Accuracy (%)
SGT	1.61	1.68	1.6	1.63	1.54	1.61	0.05	2.8%	107.5%
DVS	1.2	1.46	1.47	1.57	1.47	1.43	0.12	8.6%	95.6%
DWT	1	1.71	1.23	1.31	1.31	1.31	0.23	17.5%	87.5%
DLS	1.42	1.11	1.2	1.75	1.46	1.39	0.22	16.1%	92.5%
**Frataxin**	**1.31**	**1.49**	**1.38**	**1.57**	**1.45**	**1.44**	**0.16**	**11.3%**	**95.8%**

LQC (3.0 ng/mL)

Peptide	Replicate1 (ng/mL)	Replicate2 (ng/mL)	Replicate3 (ng/mL)	Replicate4 (ng/mL)	Replicate5 (ng/mL)	Mean (ng/mL)	SD (ng/mL)	Precision (%)	Accuracy (%)
SGT	3.11	2.87	2.92	2.97	3.09	2.99	0.09	3.1%	99.7%
DVS	2.94	2.8	3.05	2.84	2.93	2.91	0.09	3.0%	97.1%
DWT	2.92	3.29	2.58	3.28	3.09	3.03	0.26	8.7%	101.1%
DLS	2.23	3.23	3.67	3.87	3.02	3.20	0.57	17.9%	106.8%
**Frataxin**	**2.80**	**3.05**	**3.06**	**3.24**	**3.03**	**3.04**	**0.25**	**8.2%**	**101.2%**

MQC (30 g/mL)

Peptide	Replicate1 (ng/mL)	Replicate2 (ng/mL)	Replicate3 (ng/mL)	Replicate4 (ng/mL)	Replicate5 (ng/mL)	Mean (ng/mL)	SD (ng/mL)	Precision (%)	Accuracy (%)
SGT	29.52	31.96	28.83	28.06	31.8	30.0	1.58	5.3%	100.1%
DVS	28.35	29.87	30.03	29.9	29.48	29.5	0.62	2.1%	98.4%
DWT	31.82	31.6	32.58	31.52	33.19	32.1	0.64	2.0%	107.1%
DLS	32.35	32.02	30.62	32.48	31.38	31.8	0.69	2.2%	105.9%
**Frataxin**	**30.51**	**31.36**	**30.52**	**30.49**	**31.46**	**30.87**	**0.88**	**2.9%**	**102.9%**

MQC (30 g/mL)

Peptide	Replicate1 (ng/mL)	Replicate2 (ng/mL)	Replicate3 (ng/mL)	Replicate4 (ng/mL)	Replicate5 (ng/mL)	Mean (ng/mL)	SD (ng/mL)	Precision (%)	Accuracy (%)
SGT	54.99	60.07	58.46	60.85	60.47	59.0	2.15	3.6%	98.3%
DVS	47.85	52.63	57.42	57.4	56.91	54.4	3.75	6.9%	90.7%
DWT	59.35	56.74	62.04	60.17	60.37	59.7	1.73	2.9%	99.6%
DLS	58.88	57.41	61.34	61.97	60.57	60.0	1.67	2.8%	100.1%
**Frataxin**	**55.27**	**56.71**	**59.82**	**60.10**	**59.58**	**58.29**	**2.33**	**4.1%**	**97.2%**

Table S4. Inter-day precision and accuracy for determination of total frataxin in blood using 5 replicates on 3 separate days.

LQC (3 ng/mL)

Day	Peptide	Replicate1 (ng/mL)	Replicate2 (ng/mL)	Replicate3 (ng/mL)	Replicate4 (ng/mL)	Replicate5 (ng/mL)	Mean (ng/mL)	SD (ng/mL)	Precision (%)	Accuracy (%)
Day-1	SGT	3.11	2.87	2.92	2.97	3.09	3.0	0.09	3.1%	99.7%
	DVS	2.94	2.80	3.05	2.84	2.93	2.9	0.09	3.0%	97.1%
	DWT	2.92	3.29	2.58	3.28	3.09	3.0	0.26	8.7%	101.1%
	DLS	2.23	3.23	3.67	3.87	3.02	3.2	0.57	17.9%	106.8%
Day-2	SGT	3.09	2.53	2.73	2.74	2.65	2.7	0.19	6.8%	91.6%
	DVS	2.92	2.80	3.62	3.38	3.76	3.3	0.38	11.5%	109.9%
	DWT	3.02	2.66	2.94	3.47	2.59	2.9	0.31	10.6%	97.9%
	DLS	2.43	2.56	3.15	3.68	4.08	3.2	0.63	19.9%	106.0%
Day-3	SGT	2.78	2.83	2.85	1.84	1.84	2.4	0.48	19.8%	80.9%
	DVS	2.87	3.76	3.41	3.55	3.20	3.4	0.30	9.0%	112.0%
	DWT	3.26	2.58	3.40	1.68	3.00	2.8	0.62	22.2%	92.8%
	DLS	2.94	3.20	3.73	3.68	3.47	3.4	0.30	8.8%	113.5%
**Overall**	**Frataxin**	**2.88**	**2.93**	**3.17**	**3.08**	**3.06**	**3.02**	**0.35**	**11.8%**	**100.8%**

MQC (30 ng/mL)

Day	Peptide	Replicate1 (ng/mL)	Replicate2 (ng/mL)	Replicate3 (ng/mL)	Replicate4 (ng/mL)	Replicate5 (ng/mL)	Mean (ng/mL)	SD (ng/mL)	Precision (%)	Accuracy (%)
Day-1	SGT	29.52	31.96	28.83	28.06	31.8	30.0	1.58	5.3%	100.1%
	DVS	28.35	29.87	30.03	29.9	29.48	29.5	0.62	2.1%	98.4%
	DWT	31.82	31.6	32.58	31.52	33.19	32.1	0.64	2.0%	107.1%
	DLS	32.35	32.02	30.62	32.48	31.38	31.8	0.69	2.2%	105.9%
Day-2	SGT	25.36	32.54	29.67	28.2	29.98	29.2	2.35	8.1%	97.2%
	DVS	30.55	31.5	29.85	30.97	29.14	30.4	0.83	2.7%	101.3%
	DWT	29.30	28.17	30.4	29.14	29.34	29.3	0.71	2.4%	97.6%
	DLS	27.17	31.09	31.49	29.98	24.47	28.8	2.66	9.2%	96.1%
Day-3	SGT	28.24	30.28	32.72	32.25	31.54	31.0	1.61	5.2%	103.4%
	DVS	28.12	26.19	28.00	29.96	29.32	28.3	1.29	4.6%	94.4%
	DWT	29.82	31.15	32.00	29.75	30.77	30.7	0.85	2.8%	102.3%
	DLS	27.34	37.01	32.15	32.58	31.70	32.2	3.07	9.5%	107.2%
**Overall**	**Frataxin**	**29.00**	**31.12**	**30.70**	**30.40**	**30.18**	**30.28**	**1.41**	**4.7%**	**100.9%**

HQC (60 gm)

Day	Peptide	Replicate1 (ng/mL)	Replicate2 (ng/mL)	Replicate3 (ng/mL)	Replicate4 (ng/mL)	Replicate5 (ng/mL)	Mean (ng/mL)	SD (ng/mL)	Precision (%)	Accuracy (%)
Day-1	SGT	54.99	60.07	58.46	60.85	60.47	59.0	2.15	3.6%	98.3%
	DVS	47.85	52.63	57.42	57.4	56.91	54.4	3.75	6.9%	90.7%
	DWT	59.35	56.74	62.04	60.17	60.37	59.7	1.73	2.9%	99.6%
	DLS	58.88	57.41	61.34	61.97	60.57	60.0	1.67	2.8%	100.1%
Day-2	SGT	52.21	57.84	63.23	60.94	61.11	59.1	3.84	6.5%	98.4%
	DVS	59.56	57.01	58.09	58.18	60.11	58.6	1.11	1.9%	97.7%
	DWT	58.98	58.48	56.99	59.18	55.76	57.9	1.31	2.3%	96.5%
	DLS	63.52	60.4	63.58	62.53	57.55	61.5	2.29	3.7%	102.5%
Day-3	SGT	57.86	67.73	66.81	66.70	62.42	64.3	3.71	5.8%	107.2%
	DVS	58.91	65.73	55.51	55.57	55.68	58.3	3.94	6.8%	97.1%
	DWT	60.47	69.34	62.67	69.72	77.96	68.0	6.15	9.0%	113.4%
	DLS	56.66	66.50	61.50	67.93	70.39	64.6	4.92	7.6%	107.7%
**Overall**	**Frataxin**	**57.44**	**60.82**	**60.64**	**61.76**	**61.61**	**60.5**	**1.57**	**5.0%**	**100.8%**

Table S5 Comparison of calibration curves in 5% BSA and in FA blood

Peptide	Slope 5% BSA	Slope FRDA blood	Mean	CV (%)
$S^{81}GTLGHPGSL^{90}$	0.07598	0.07913	0.07756	-4.1%
$D^{124}VSFGSGVLTVKLGG^{138}$	0.09619	0.1081	0.10215	-11.7%
$D^{157}WTGKNWVYSH^{177}$	0.05700	0.05959	0.0583	-4.4%
$D^{199}LSSLAYSGK^{208}$	0.04467	0.04731	0.04599	-5.7%

CV: coefficient variation

Table S6. Stability of total frataxin, mature frataxin and frataxin isoform E in whole blood (controls, N=3) before sample process over 24 hr on the benchtop.

	Total frataxin (ng/mL)			Stability (%)		
Hours at RT	Control 1	Control 2	Control 3	Control 1	Control 2	Control 3
0	37.48	45.12	35.92			
1	34.42	44.81	35.61	91.84%	99.30%	99.13%
2	38.57	47.62	38.72	102.91%	105.54%	107.80%
3	42.62	44.12	35.42	113.71%	97.78%	98.61%
4	41.06	43.53	34.23	109.55%	96.47%	95.29%
6	39.38	50.00	41.00	105.07%	110.81%	114.13%
8	38.48	44.37	30.87	102.67%	98.34%	85.94%
12	45.42	51.57	42.17	121.18%	114.30%	117.41%
24	45.68	48.42	39.62	121.88%	107.31%	110.29%

	Mature frataxin (ng/mL)			Stability (%)		
Hours at RT	Control 1	Control 2	Control 3	Control 1	Control 2	Control 3
0	6.20	7.84	6.99			
1	6.70	7.96	6.96	108.01%	101.51%	99.55%
2	6.06	8.41	7.41	97.67%	107.27%	106.01%
3	7.45	7.64	6.64	120.14%	97.45%	95.00%
4	5.73	7.69	6.69	92.41%	98.08%	95.71%
6	6.29	8.29	7.29	101.43%	105.72%	104.28%
8	6.13	8.10	7.10	98.83%	103.24%	101.49%
12	6.25	9.73	8.73	100.85%	124.05%	124.83%
24	6.52	8.20	7.20	105.09%	104.63%	103.05%

	Frataxin isoform E (ng/mL)			Stability (%)		
Hours at RT	Control 1	Control 2	Control 3	Control 1	Control 2	Control 3
0	31.28	37.28	28.93			
1	27.72	36.85	28.65	88.63%	98.84%	99.02%
2	32.51	39.21	31.31	103.95%	105.18%	108.23%
3	35.17	36.48	28.78	112.44%	97.85%	99.48%
4	35.33	35.84	27.54	112.95%	96.13%	95.19%
6	33.09	41.71	33.71	105.79%	111.88%	116.52%
8	32.35	36.27	23.77	103.43%	97.31%	82.18%
12	37.17	41.85	33.45	118.82%	112.25%	115.62%
24	36.16	40.21	32.41	115.62%	107.87%	112.04%

stability (%)= Concentration of frataxin (hour)/ concentration of frataxin (T=0) %

Table S7. Stability of frataxin after two cycles of free-thaw at MQC (30 ng/mL, n=3) (A) Fresh blood. (B). Blood after two freeze-thaw cycles.

A

Sample	SGT (ng/mL	DLS (ng/mL	DWT (ng/mL)	DVS (ng/mL)	DET (ng/mL)	Mature (ng/mL)	Isoform E (ng/mL	Total (ng/mL)
R1	33.2	34.7	35.9	37.2	34.3	33.2	3.3	36.5
R2	33.4	39.6	36.3	36.2	33.8	33.4	2.8	36.2
R3	32.8	31.6	35.9	35.3	36.0	32.8	2.8	35.6
Mean (ng/mL)	33.1	35.3	36.0	36.2	34.7	33.1	3.0	36.1
SD	0.3	4.1	0.2	0.9	1.2	0.3	0.3	0.5
RSD	1.0%	11.5%	0.5%	2.6%	3.3%	1.0%	9.5%	1.3%

B

R1	28.3	26.1	34.1	27.6	31.7	28.3	2.6	30.9
R2	30.6	24.0	32.1	34.2	32.1	30.6	2.5	33.1
R3	29.9	30.8	33.8	32.7	33.6	29.9	3.4	33.2
Mean (ng/mL)	29.6	27.0	33.3	31.5	32.5	29.6	2.8	32.4
SD	1.2	3.5	1.1	3.4	1.0	1.2	0.5	1.3
RSD	4.1%	13.0%	3.2%	10.9%	3.1%	4.1%	16.3%	4.1%

Table S8. Incurred sample reanalysis in twelve FA whole blood samples

Incurred samples reanalysis (N=12) for mature frataxin in whole blood

Subject	First analysis (ng/mL)	Repeated analysis (ng/mL)	Mean (ng/mL)	Deviation (%)
FA_01	2.01	2.14	2.07	6.70%
FA_02	2.23	2.32	2.27	4.15%
FA_03	1.15	0.95	1.05	-19.50%
FA_04	2.76	2.61	2.69	-5.69%
FA_05	1.66	2.00	1.83	18.66%
FA_06	1.44	1.68	1.56	15.26%
FA_07	3.11	2.67	2.89	-15.44%
FA_08	1.69	1.75	1.72	3.47%
FA_09	2.59	2.90	2.75	11.09%
FA_10	1.92	1.61	1.77	-18.01%
FA_11	3.29	3.05	3.17	-7.63%
FA_12	4.22	3.80	4.01	-10.32%
Average	2.34	2.29	2.31	-2.16%

Incurred samples reanalysis (N=12) for frataxin isoform E in whole blood

Subject	First analysis (ng/mL)	Repeated analysis (ng/mL)	Mean (ng/mL)	Deviation (%)
FA_01	7.36	8.56	7.96	15.16%
FA_02	6.07	5.25	5.66	-14.46%
FA_03	5.22	4.35	4.79	-18.19%
FA_04	14.84	12.77	13.80	-14.98%
FA_05	4.07	4.73	4.40	15.17%
FA_06	4.29	3.97	4.13	-7.69%
FA_07	13.53	10.35	11.94	-26.62%
FA_08	6.63	6.03	6.33	-9.55%
FA_09	9.20	8.45	8.82	-8.49%
FA_10	9.21	7.91	8.56	-15.24%
FA_11	11.50	8.99	10.24	-24.52%
FA_12	14.56	11.87	13.22	-20.36%
Average	8.87	7.77	8.32	-13.26%

Incurred samples reanalysis (N=12) for total frataxin in whole blood

Subject	First analysis (ng/mL)	Repeated analysis (ng/mL)	Mean (ng/mL)	Deviation (%)
FA_01	9.36	10.71	10.04	13.41%
FA_02	8.29	7.57	7.93	-9.13%
FA_03	6.37	5.30	5.84	-18.42%
FA_04	17.60	15.38	16.49	-13.46%
FA_05	5.73	6.74	6.23	16.20%
FA_06	5.73	5.65	5.69	-1.40%
FA_07	16.64	13.01	14.83	-24.44%
FA_08	8.32	7.77	8.04	-6.77%
FA_09	11.79	11.35	11.57	-3.84%
FA_10	11.14	9.51	10.33	-15.72%
FA_11	14.79	12.03	13.41	-20.53%
FA_12	18.78	15.67	17.23	-18.02%
Average	11.21	10.06	10.63	-10.84%

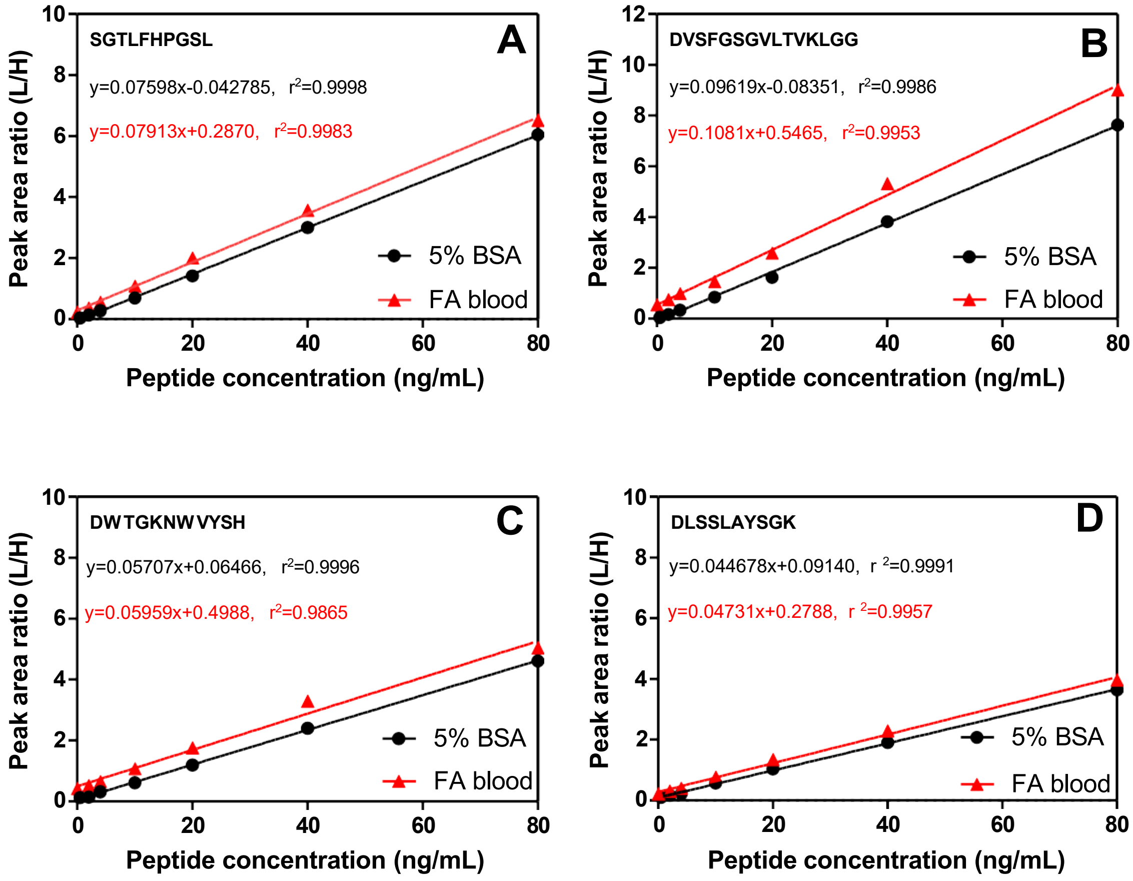

Figure S1. Calibration curves constructed in 5% BSA (black) and a whole blood pool from seven FA patients (red) in the range of 0.5 ng/mL to 80 ng/mL. (A) S^{81}GTLGHPGSL^{90}, (B) D^{124}VSFGSGVLTVKLGG^{138}, (C) D^{157}WTGKNWVYSH^{177}, (D) D^{199}LSSLAYSGK^{208} were plotted against peptide concentration (ng/mL). The slope, intercept and regression coefficient (r^2) are shown in each figure.
